# Medical professionals’ agency and pharmaceuticalization: Physician-industry relations in Russia

**DOI:** 10.1177/13634593221116508

**Published:** 2022-08-01

**Authors:** Ekaterina Borozdina, Olga Zvonareva

**Affiliations:** European University at St. Petersburg, The Russian Federation; Siberian State Medical University, The Russian Federation; Maastricht University, The Netherlands; Siberian State Medical University, The Russian Federation

**Keywords:** agency, industry, knowledge, medical professionalism, pharmaceuticalization

## Abstract

In the contemporary world pharmaceuticals have become a go-to answer to a growing number of questions. This process of pharmaceuticalization gives rise to a concern with the increasing influence of the pharmaceutical industry on physicians’ decision-making. Critics suggest that companies’ for-profit-interests might compromise the integrity of medical practice. This article employs qualitative research methodology to explore how Russian physicians deal with the industry’s efforts to expand and shape the use of pharmaceuticals. By bridging perspectives of social studies of science and sociology of professions, we offer a contextualized account of physicians’ daily practices and interpretations related to pharmaceuticalization. The findings question conventional assumptions of physician-industry relations and allow to delineate a new form of medical professionalism that emerges in the context of pharmaceuticalization and cannot be reduced to either “resisting” industry marketing activities or “giving in” to them and thus corrupting biomedical expertise. Instead, the ways in which physicians navigate abundant sources of knowledge and use industry resources to overcome constraints of their organizational environment attest to mundane forms of agency exercised by physicians in their relations with industry.

## Introduction

Recognition of the rising importance and the novel roles of pharmaceuticals in society has been reflected in the scholarship on pharmaceuticalization. Pharmaceuticalization involves “the transformation of human conditions, capabilities and capacities into opportunities for pharmaceutical intervention” (Williams et al., 2011: 711). It has been intensifying in recent decades as new markets and health conditions for pharmaceutical interventions have been established alongside scientific advances in biomedicine that enable the development of novel pharmaceuticals.

Among the influential drivers of pharmaceuticalization is the promotion by pharmaceutical companies targeting physicians and members of the public (Abraham, 2010). While historically, autonomous and powerful medical profession has dominated clinical setting (Freidson, 1970), currently leading role of pharmaceutical industry in biomedical knowledge production and dissemination poses a challenge to this order. Some even argue that the medical profession has ceased to be the main driver of the medicalization process, as the industry intercepts this role, at least in western societies contributing to the increase and change of pharmaceuticals use beyond medicalization (Abraham and Lewis, 2002; Clarke et al., 2003).

Despite these concerns, the role of medical professionals in the pharmaceuticalization process remains insufficiently studied empirically. In this article we address this gap by answering the question of how physicians engage with complex knowledge and organizational landscapes produced by pharmaceuticalization. Two existing bodies of literature are directly relevant to our undertaking—social studies of pharmaceutical knowledge and studies of medical professionalism.

Studies of pharmaceutical knowledge devote considerable attention to the various ways in which the public engages with the pharmaceuticals made available and promoted by the industry. Some studies focus on the patient groups’ activities to access specific drugs and/or to challenge practices of biomedical research (Brown et al., 2004; Epstein, 1996). Others highlight that individuals may be reluctant “pharmaceutical consumers,” who prefer to minimize drug use, following their own rationales (Will and Weiner, 2015). Overall, it has been suggested that in the everyday life of people, pharmaceuticals obtain contextual, social, and symbolic meanings, become tied to identities, and raise moral concerns; all shaping diverse patterns of their use which cannot be reduced to such dichotomies as active consumption/resistance (Dew et al., 2015). At the same time, when empirical attention turns to investigating how physicians deal with the industry, the analytical focus is mostly placed on the “active consumption” of the industry promotional efforts. Studies have documented elaborate strategies of pharmaceutical representatives to manipulate physicians’ prescription behavior and emphasized physicians’ susceptibility to these strategies (Fugh-Berman and Ahari, 2007), with less attention to the strategies and agency of physicians themselves.

Studies of medical professionalism, in turn, view doctors as actors embedded in an institutional landscape characterized by power struggles between different healthcare professions, patients, state authorities, and pharmaceutical companies. Pharmaceuticalization is considered to be a crucial challenge to the medical profession as it allows industry to wrestle the control over knowledge production from physicians and thus to undermine their autonomy and authority (Light, 2010; Timmermans and Oh, 2010). However, while focusing on rivalry and agency within the medical domain, professionalism scholars with a rare exception (Timmermans and Angell, 2001) avoid exploring the content of the physicians’ tasks and pragmatics of clinical decision-making shaped by the medical knowledge about pharmaceuticals.

In summary, when analyzing the role of medical professionals in pharmaceuticalization, studies of pharmaceutical knowledge tend to focus on physicians being influenced by the pharmaceutical industry and industry-produced knowledge about medicines. The agency of physicians is rarely the focus. Studies of the medical profession primarily concentrate on the issues of power and discuss physicians’ agency but tend to bracket the influences of pharmaceutical knowledge on the daily practices of physicians.

We draw on these two lines of academic discussion to provide a nuanced account of medical professionals’ activities in the context of pharmaceuticalization. We employ qualitative data from Russia to explore ways in which physicians shape their relations with the representatives of pharmaceutical companies and deal with the efforts of the latter to expand and shape the use of drugs.

## Physicians and industry: Corruption and agency

Concerns about the physician-industry relationships stem from the possibility of conflicts of interest between the physicians’ duty to their patients and their potential obligations to pharmaceutical companies. Companies attempt to influence the attitudes and prescription behavior of physicians through the regular visits of pharmaceutical representatives, advertisements in professional venues, and gifts, from inexpensive “reminder” items to sponsoring education and travel to conferences (Padamsee, 2011). Some of the available literature focus on the ethical implications of such interactions (Marco et al., 2006) and surveying corresponding attitudes of physicians (Brett et al., 2003); the strategies employed by companies to enter medical practice (Fugh-Berman and Ahari, 2007); and the extent of industry’s influence on physicians’ decision-making (Wazana, 2000). Several qualitative studies explored physicians’ accounts of how and why they interact with the pharmaceutical industry (Chimonas et al., 2007; Doran et al., 2006; Prosser and Walley, 2003). Many authors in this topic to some extent adopted a view of a strict separation of the industry with its marketing efforts, and the clinic with its rational pharmacotherapy. Physicians are viewed as those whose integrity needs to be guarded by normative guidelines and regulations, with some authors offering the option to ban physician-industry interactions (for instance, Chimonas et al., 2007).

However, critical social studies highlight how marketing and biomedical expertise are not clearly separate but closely entangled (Lakoff, 2004; Wadmann, 2014). Recent publications on the medical professionalism also argue that a more nuanced approach is needed to understand contemporary role of physicians—an approach that not only recognizes boundaries within healthcare but theorizes physicians’ ability to maintain discretion while being connected to other actors in a complex clinical environment (Noordegraaf, 2020). In this article we continue these lines of argument and employ a neo-institutional view on professional agency to analytically connect the behavior of individual physicians with the structural conditions of healthcare organizations.

While organizational studies commonly depict institutions (healthcare included) as cognitive, normative, and regulative structures that give meaning to social behavior (Scott et al., 2000: 167), current scholarship supplements this macro-level view with the understanding of how institutional processes operate from the ground up. This line of theorizing known as “inhabited institutionalism” (see for a review, Hallett and Hawbaker, 2021) portrays institutions as reproduced and changed through the meaning-making activities of those individuals who populate organizational setting. It provides us with two insights that are indispensable for a comprehensive analysis of physician-industry relations.

First, by framing individual agency as a derivative of mundane organizational interactions (Hallett and Ventresca, 2006), this approach departs from the view of professionals as hypermascular actors who uphold their autonomy and powerful position by resisting extraneous influences. Instead, it focuses on physicians’ routine activities that are shaped by and constitutive of institutional arrangements. Such activities do not lead to dramatic shifts, but they subtly contribute to the introduction and maintenance of meanings, identities, and scripts for action in the clinical setting (Lawrence et al., 2009).

Second, this approach postulates that organizational complexity does not restrain, but stimulates professional agency (Smets and Jarzabkowski, 2013). Modern healthcare constitutes a pluralistic institutional environment, where different stakeholders (including professionals, patients, governmental bodies, and businesses) with their distinctive values and interests come at crossroads. The necessity to manage this complexity in search for an optimal balance between contradictory logics and principles gives rise to—a somewhat compelled—ground-level agency of medical professionals.

Russian healthcare constitutes a particularly instructive case to study such agentic response of physicians to a pharmaceutical industry. In this organizational context, the doctors, who have already endured interventions of a powerful national government (Saks, 2015), are facing the task to reconcile professionalism, bureaucratization, and the novel challenge of pharmaceuticalisation. In what follows, we discuss in detail how, by managing relations with the industry and its pharmaceutical representatives, physicians in Russia construct additional ways of reconfiguring medical expertise and establishing their professional identity. This discussion cannot be used for broad generalizations regarding physicians’ opinions and practices. But it sheds light on mundane forms of agency through which physicians enact their professionalism and preserve integrity of clinical practice under challenging circumstances of pharmaceuticalization.

## Data and methods

This paper relies on two sets of qualitative data. The first set of data was collected as a part of a research project concerned with knowledge, evidence and credibility in Russian cardiovascular medicine conducted in 2014–2016. It involved an analysis of publicly available professional discussions on Rusmedserv, one of the oldest and most popular medical forums used by Russian physicians; analysis of relevant policies, primarily law No. 323-FZ “On public health protection in the Russian Federation” which regulates relationships between clinics and pharmaceutical industry; and in-depth interviews with 15 cardiologists in a large city in the Western part of Russia. Informants were recruited using the snowball technique, with the initial informants being identified through a state research center specializing in cardiovascular medicine. All 15 informants were cardiologists with two working as general practitioners; all but one informant worked in state-funded facilities: primary healthcare facilities (5), out-patient departments of hospitals (2), and in-patient departments of hospitals (8). Their professional experience ranged from 1 to 2 years to almost 30 years of medical practice.

The second set of data was collected in 2018–2019 as a part of a research project on social aspects of clinical trials, including the relations between trial findings and actual clinical practice. This time 25 in-depth interviews with general practitioners were conducted in a major city in Eastern part of Russia. All the informants worked in state-funded medical facilities: primary care facilities (18) and hospitals (7). Half of the informants (13) worked in healthcare for less than 5 years, and another half represented those who have worked for more than 5 years. The minimal clinical experience in the sample was 6 months, the maximum—40 years of work. The contacts of a few initial interviewees were provided to the researchers by a local medical university, subsequent informants were recruited using a snowball sampling. Both samples cannot be considered representative and therefore, our results do not provide information about how wide-spread certain views and practices are. They are, however, indicative of a range of professionals’ responses to pharmaceuticalization in different organizational environments within Russian healthcare –healthcare facilities in major city characterized by better access to resources and educational opportunities and more typical case of medical organizations in an “average” Russian city.

Interviews in both projects explored how physicians navigate abundant new information in their fields, how they approach gaining new knowledge, which information sources they trust and why, how they assess new information, and how they decide whether to use it in their practice. The topic of industry, its promotion efforts and the physicians’ communication with pharmaceutical representatives featured prominently in all informants’ accounts. During the interviews we more specifically asked informants for details of their interactions with industry, their motivations, and considerations. The interviews were audio-recorded, transcribed verbatim, and anonymized. The interviewees were provided with information about the study and gave their oral consent to participate. Both projects were approved by the ethical committee of The Russian National Medical Research Center for Preventive Medicine.

Interview data were analyzed using the version of thematic analysis (Deterding and Waters, 2018). First, the interview transcripts were indexed, linking their content with the key analytical categories employed in this study – physicians’ agency, the organizational context of physicians’ work, professional identity, and medical expertise. Second, the identified parts of interviews were coded using the codes derived from the data, which helped to empirically imbue (and sometimes to challenge) the initial research assumptions. For example, many informants spoke at length about critical assessment of information shared by pharmaceutical representatives and their detailed accounts provided an opportunity to distinguish and employ in the coding process specific strategies such as checking foreign guidelines and assessing reputation of a particular pharmaceutical company. The analytical categories served to organize codes. For example, the code difficulties in accessing information was included in the organizational context of physicians’ work and the code physicians as gatekeepers became a part of professional identity. Analysis of the online forum discussions focused on how physicians assessed the possibility of conflict of interest between their duty to patients and potential obligations to pharmaceutical companies that the law No. 323-FZ was meant to prevent. Regular critical discussions with other members of the project teams were held to enhance the interpretive rigor and the quality of the analysis.

## Russian health system: Professional autonomy, institutional reforms, and evidence

Since the dissolution of the Soviet Union, two contradictory policy trends conditioned transformations of the Russian health system – statism and neoliberalism (Kulmala et al., 2014; Matveev and Novkunskaya, 2022). The former trend continues the Soviet legacy of strict state control and centralized funding of health care. The latter trend is reflected in the attempts to reorganize healthcare by introducing market incentives and competition in the provision of medical services. While unfolding within the system of centralized and publicly owned medical facilities, these trends have defined the institutional conditions for clinical decision-making.

Most physicians within this system are salaried state employees. Studies indicate that they are overburdened, bogged-down in bureaucratic work, and lack time for professional development, since governmental aspirations for maintaining universal care go hand in hand with extensive administrative control and are not backed up with sufficient funding (Matveev and Novkunskaya, 2022; Sheiman et al., 2018). Since the 1950’s sociologists have been questioning the degree of professional autonomy that is possible under these conditions. The most radical assumptions equate (post-)Soviet physicians with minor bureaucrats who are deprived of professional discretion and function as passive executors of the administratively defined goals (Field, 1957). Others admit that while the ability of (post-)Soviet physicians to influence the conditions of their work is significantly limited, they possess a certain degree of technical autonomy in performing their duties (Freidson, 1970: 23–70; Saks, 2015).

Marketization that has entered the Russian health system in the aftermath of state socialism, constituted an additional challenge to the medical professionals. Rapid neoliberal reforms resulted in the fragmentation of the country’s healthcare (Cook, 2014), with the new funding schemes aggravating disparities between medical facilities in terms of available equipment and physicians’ access to continuous training. Emerging consumerism constituted yet another challenge to medical professionals. Patients became more captious regarding the quality of the medical services: some started to “shop around” in search of a better specialist, while the others use administrative and legal pathways to penalize the clinic and physician for low quality treatments (Litvina et al., 2020; Temkina, 2020).

Arrival of evidence-based medicine (EBM) ideas to the country contributed to yet another level of ambiguity regarding physicians’ professional autonomy and authority. In Soviet healthcare the EBM paradigm was a subject of ideological critique as unethical and capitalist—one that prioritizes not the pragmatics of clinical tasks, but abstract scientific principles, and corresponds to for-profit interests of pharmaceutical companies rather than to the public good (Vasilyev et al., 2021). While it was welcomed in Russia in the 1990s on the wave of healthcare internationalization, its actual implementation in clinical settings was impeded by institutional obstacles, including lack of material resources, outdated medical educational programs, and rigid bureaucracy (Geltzer, 2009). Moreover, standardization of medical practice with the reference to the EBM principles was mostly performed by the state regulatory bodies, not by the medical community. This provoked distrust of individual physicians to the governmentally approved standards since they doubted the scientific validity of these documents and perceived them as yet another vehicle of bureaucratic control over the medical profession (Kamenshikova, 2018).

Pharmaceutical companies entered this volatile landscape in the early 1990s, bringing conventional marketing practices, including the work of pharmaceutical representatives as a key element of establishing relations with physicians. For a while, the relationships between the industry and health care were barely regulated. The norms in the medical community—members of which suddenly found themselves in a changed environment—and in the newly emerged pharmaceutical marketing community regarding what could be acceptable ways of engaging with each other, were being explored. In the context of the underfunded healthcare system, these ambiguities allowed for the existence of a wide range of engagement strategies, including the far from innocent ones (Vlassov et al., 2001).

The regulatory attitudes toward the intersection of the pharmaceutical industry and clinical practice markedly swung again in 2012. By 2012, from a state of nonexistence, the Russian pharmaceutical market grew to be the seventh largest in the world (DSM Group, 2012: 3) and healthcare organizations developed routines for interacting with industry representatives, while individual physicians had the time to orient themselves in the market environment and to choose their stance regarding relations with the industry. To ensure the integrity of physicians and protect proper medical practice, a law No. 323-FZ “On public health protection in the Russian Federation” that came to force in November 2012, contained Article 74. Echoing discussions in the media and prior statements made by concerned state officials, this article prohibited “medical workers and the heads of medical institutions” to receive gifts and money from pharmaceutical companies (clinical trials are not covered by this prohibition). It also stipulated that they may not meet pharmaceutical representatives at the workplace apart from instances of “participation in medical staff meetings and other events aimed at [staff’s] performance improvement or at presenting information on monitoring medicines” safety or medical products’ safety, all according to the procedure stipulated by the administration of the medical organization’ (Article 74.1.5). It is after this reform that this study was carried out to analyze current ways in which physicians shape their relationships with the pharmaceutical industry.

## Pharmaceutical representatives in Russian medical organizations

While new legislation did cause turmoil, after a while pharmaceutical representatives’ visits to clinics resumed with little change from before the enactment of law No. 323-FZ. In 6 months after the enactment of the law a large social network for Russian medical professionals “Doctor at work” undertook a poll among its members. According to the results, around 18% of medical professionals reported that individual visits “almost ceased” and the rest continued receiving pharmaceutical representatives, albeit some more rarely (Maskina, 2012). Our study shows that 7 years after the introduction of the law, talks to pharmaceutical representatives still constitute a part of the Russian doctors’ routine. Interview materials also provide an insight about how the organizational features of Russian healthcare—bureaucratization and regional inequalities between medical facilities—condition continuous pharmaceutical representatives’ presence.

When describing their work routine, our informants name several ways in which state bureaucratic control over healthcare impedes the acquisition and usage of new biomedical knowledge. First, they stress limited possibilities for continuous medical education. One of the informants describes how within bureaucratized health system such education turns into yet another burdensome formality:

Speaking of continuous education, we have a specialist in the personnel department who simply makes us. . . she gives us vouchers, and we are obliged to undergo this training in [the name of the university]. I will be in this training for the second time, but you know, it is a distance learning. To be honest, I would like to have a full-time off-the-job training. But we do not have such an opportunity. This is a big problem, because [. . .] I probably spent three days, actually every evening until two in the morning just completing the tests. (GP1)

Furthermore, another informant, a cardiologist who is employed in a state hospital and is also working on a PhD dissertation in a research institute, explained that ineffective bureaucracy hinders dissemination of new treatment guidelines and recommendations. In such situation, pharmaceutical representatives become one of the few functioning, albeit far from ideal, ways of receiving updates regarding novelties in drugs and clinical trials:

I don’t know, maybe everything needs to be systematized . . . So, perhaps, that these general guidelines are sent to hospitals, to polyclinics. They are not disseminated in this way now. Only if a pharmaceutical representative comes to us, gives us something. But they would give us [information] only about their own drug. While we need to have a big picture. Or now I came here [a research institute] and got this pile of recommendations. Good, but I got it here, in the institute. They did not reach a polyclinic or our hospital, so. . . Also, not everyone reads things on Internet or in journals. There is a need to disseminate information more adequately, so that it reaches everyone. (Cardiologist8)

Informants conveyed a manner of engaging with pharmaceutical representatives which was somewhat akin to watching the daily news: representatives’ “channel” delivers new information quickly, but if one needs comprehensive and analytical information other sources have to be consulted. The representatives perform a “signaling” function, alerting physicians to the availability of new pharmaceuticals on the market or new indications and treatment regimens for already established drugs.

Physician-representative interaction maps into the country’s health system, including regional inequalities. Some of our informants worked in a large city in the Western part of Russia, where multiple scientific and educational events are held, and many medical workplaces have computers and Internet access. Outside such cities the situation with physicians’ access to new and relevant information may be hampered to a larger extent. As one of the informants narrated their experience of communicating with physicians from Shatura, a town 138 km from Moscow:

Pharmaceutical representatives are, perhaps, central. They travel even to Kolomna, to Shatura, even to remote places, regions, even in oblast [area around Moscow]. We accidentally met doctors from Shatura, somewhere during a conference. And they told us that if there were no pharmaceutical representatives, then they would not know anything at all. Because it is almost impossible to regularly go to Moscow from Shatura. 3 hours by train. (Cardiologist8)

Overall, pharmaceutical representatives form an important channel of pharmaceutical news delivery compensating for institutional deficiencies including lack of access to information about current developments in pharmaceuticals within the bureaucratized health system. Almost all interviewed physicians pointed to the efficiency of pharmaceutical representatives’ work: it appeared convenient that the representatives brought information directly to their workplace, including those workplaces that are located far away from regional centers. These perspectives correspond with the results of a poll administered by the Russian Public Opinion Research Centre in 2010 among physicians, where respondents indicated that the main sources alerting them to the appearance of new pharmaceuticals are medical conferences and congresses (72%) and visits of medical representatives (71%) (AIPM and WCIOM, 2010).

However, physicians were aware that the “news” delivered by the pharmaceutical representatives is not comprehensive and might be biased. Rather this “news” stimulates physicians to obtain more detailed information about new drugs from a variety of other sources.

## Enacting professionalism in the interactions with pharmaceutical representatives

One of the key challenges that contemporary medical professionals face is the task of preserving control over the content of their work while being increasingly exposed to market and bureaucratic influences. In what follows we explore how Russian physicians reconcile the autonomy of clinical decision-making, and their actual connectedness to various actors and sources of knowledge within the changing institutional environment. We do this by focusing on the two processes that take place within the clinical routine: (1) the reconfiguration of the professional expertise and (2) the construction of professional identities. Neo-institutional scholars depict these two processes as crucial vehicles through which professional and institutional dynamics are connected (Suddaby and Viale, 2011). Attention to them allowed us to delineate forms of mundane agency which are exercised by physicians in their relations with industry in the context of pharmaceuticalization.

### Reconfiguring professional expertise

Possession of biomedical knowledge lies at the heart of modern medical professionalism. One of the early sociological definitions portrays a physician as a person able to make a connection between the abstract scientific expertise and the idiosyncrasies of a patient’s experience (Freidson, 1970). Our research also suggests that medical professionalism is about physicians’ ability to establish connections, but not only between “pure” knowledge and “messy” reality, but also between different kinds of knowledge. While noting the efficiency of pharmaceutical representatives, the doctors stressed that they do not blindly rely on what is being delivered to them by the industry, but reflectively consider and pragmatically adjust this knowledge to particular organizational environment. The informants conveyed that the information from individual pharmaceutical representatives is often general and of a surface level and while it has its place among their ways to navigate in the abundance of pharmaceutical data, it is not given a major role:

Information from companies is little and surface-level. Maybe, some people who provide it have some medical education, some were trained as pharmacists, there are such pharmaceutical representatives. But generally, these are people without medical education, they can just give memorized statistical data. . . . Also, I cannot trust their data, they don’t always know what they are talking about. There are some memorized texts and that’s it. . . . There are some questions and, when a person has at least pharmacist training, it is already easier to talk to them. But there are few of them. (Cardiologist1)

Social science scholars suggest distinguishing two types of medical professionalism—“traditional” and “modern”—depending on what source of knowledge physicians prioritize in their practice (Timmermans and Berg, 2003: 88–89). “Traditional” professionalism is grounded in clinical experience and collegial decision-making within professional community, “modern” professionalism predominantly relies on formal documents such as clinical guidelines and standards of medical practice. Our findings indicate that physicians actually oscillate between these two types of professionalism and exercise agency by building connections between the knowledge provided by medical representatives, on the one hand, and clinical standards and advices of colleagues, on the other hand.

Interviewees reported employing a variety of criteria for assessing whether the drug promoted by the pharmaceutical representative is worth using in clinical practice. Recognizing the limitations of the pharmaceutical representatives’ “news channel,” physicians practice searching for additional information, cross-checking sources. While many physicians reported looking for professional guidelines and checking in the available ones whether the promoted drug is mentioned there, the others stated that their colleagues’ opinions regarding particular new drugs are most significant for them.

In line with the generally conveyed strategy of cross-checking, none of the interviewed professionals singled out a particular source that would be considered sufficient for obtaining reliable information. Physicians compare the data from different sources and describe being inclined to wait for an accumulation of a “critical mass” of congruent opinions and references to a new drug before bringing it into their practice:

In any case, I check all information in sources, in different medical publications, look at reviews on medical websites, and in the European and US guidelines. (Cardiologist5)

Informants also reported assessing the reputation of the pharmaceutical company that produces the drug more generally—how established and invested it is in clinical trials and drug development. Physicians expressed more trust in drugs of well-known companies that run their own clinical research. The excerpt below shows how a physician combines this reputational method of assessment with analysis of their own clinical experience:

We are looking at a pharmaceutical company. There are pharmaceutical companies that have proven themselves better in the market, and there are some new, say, not yet promoted pharmaceutical companies. Additionally, [we rely on our] clinical experience. That is, the drug of some pharmaceutical company . . . if the patient was taking the drug, and the effect was good, then we trust this pharmaceutical company more. Well, this is a kind of comparative analysis. (GP5)

Furthermore, physicians think about their relationships with the pharmaceutical representatives and companies in a long-term fashion and take account of the reputation companies acquired in their eyes. Prior interactions as well as the quality of previously supplied marketing materials affect how physicians view further attempts to engage with them. For instance, one cardiologist described how a previous experience of communicating with a company has led to their reluctance to use the company’s products in the future:

There is a company XX. They are very actively introducing one drug, Y, into practice. Visited several times. There are many sartans, this is one more of these. But they did not bring any convincing data, why I should prescribe exactly their sartan. There need to be at least some arguments. And also, honestly, I am not very eager to hear arguments of this company. Once I caught them on not entirely honest promotion of a drug. They gave a brochure, comparative efficacy, where drugs being compared were taken by them in dosages which were certainly below therapeutic ones. For an idiot who will read conclusions and won’t look at the design, in which dosages drugs are compared. (Cardiologist4)

Overall, the messages brought by pharmaceutical representatives prompt physicians to search for additional information and engage in reflexive information processing, drawing on a variety of sources, and taking the representatives’ “news” as a starting point for further explorations. Physicians consider it their professional responsibility to check these incoming messages and are increasingly confronted with the need to tie together different sources and types of information to be able to perform this responsibility well.

### Constructing professional identity

The normative core of medical professionalism is associated with the prioritization of the quality of medical service and of patients’ interests (Scott et al., 2000: 179–181). Pharmaceuticalization threatens this hierarchy of values as advertising efforts of the industry frequently bring to the fore not professional, but market rationale. Professionals’ identity work is instrumental for reconciling these competing institutional pressures (Bévort and Suddaby, 2016).

Our informants describe a complex web of relationships which physicians navigate and use industry resources to overcome the constraints of their institutional environment rather than committing outright moral breaches. When critically discussing marketing practices of pharmaceutical companies’, physicians construct an image of medical professional who engages with the industry without necessarily compromising their integrity. For instance, one interviewed cardiologist stressed a critical stance of medical professionals toward the pharmaceutical industry, granting physicians power and agency that appears to be stripped away in the context of extensive state control and bureaucratization:

It is a pity that pharmaceutical representatives are being restricted now, are being made look evil. Actually, it is very difficult to force a physician to do something, a physician will act the way she deems appropriate, regardless. It is also difficult to really convince a physician. Physicians still more look at, say, professors, rather than pharmaceutical representatives. (Cardiologist4)

The Russian medical community largely appears to draw a difference between engaging with the industry and “selling out,” that is promoting and prescribing whatever a pharmaceutical company offers. Many physicians disagree with the necessity of limiting what came to be seen by them as legitimate information delivery arrangements. An illustration can be provided by a discussion regarding relationships between physicians and pharmaceutical representatives that happened within a large Russian medical online forum back in 2006. With a few medical professionals accusing the local medical community of “jumping through hoops” for pharmaceutical companies, which indicates a diversity of opinions on the matter among physicians themselves, most maintained that there are ways to professionally interact with the industry without being labeled as “selling out”:

Thank heaven I know how to treat hepatitis myself, without pharmaceutical representatives telling me (you know, I studied, abroad as well), but if a pharmaceutical representative brings me a new article from Hepatology or New England Journal of Medicine (I don’t have a subscription, unfortunately) – what is wrong with it? And if I know where to send a patient for one or another drug and am sure that in that pharmacy PegIntron or ribavirin or something else that I prescribe is authentic and not counterfeit and a price there will be the best in the city – what is wrong with it? (Infectious disease specialist1, online forum discussion)

Indeed colleagues, we all . . . live in a society without well-established ethical norms. All of us – both doctors, irrespectively of whether they work in private or public medicine, and [medical] teaching staff – regularly encounter attempts to be bought. And let’s be honest, there are colleagues who do sell out. But I do not consider this to be a reason to doubt the integrity of the majority of physicians, collaborating with representatives of pharmaceutical companies. And consider such categorical judgments unjustified. (Medical education specialist1, online forum discussion)

By rejecting the charge of being manipulated or corrupted by contacts with pharmaceutical representatives, physicians assert their professionalism in a particular way. Previous studies have already highlighted that physicians’ identity work can be performed through engaging with managerial interventions within contemporary healthcare (Correia, 2017; Kyratsis et al., 2017). This study adds that the practice of pragmatic reconciliation of different kinds of knowledge, including information delivered by pharmaceutical representatives, becomes not just the prerequisite of successful clinical practice but also constitutes the foundation of physicians’ professional identity.

Our informants consider the ability to navigate and connect different sources of knowledge in order to guide clinical decision-making as central to contemporary medical professionalism. In this reasoning excessive influence of pharmaceutical representatives exerted on some physicians is not denied. It is acknowledged but understood as a consequence of the insufficient ability of some medical practitioners to make clinical decisions through skillful maneuvering within a complex knowledge environment made additionally rocky by institutional constraints:

Prescription of antiviral drugs for any viral infections, which is absolutely not proven [to be effective]. . . [The names of the drugs] are all a kind of homeopathy that doesn’t work. But there are paramedics and older doctors, they seem to recommend this without any hesitation, just because they were told by the pharmaceutical representatives ‘here is such a drug, research was done, etc., etc..’ And then you explain [to them] that there is a problem with those studies (. . .) that there is no statistically significant difference [between the drug and the placebo]. (GP8)

Thus, physicians who adopt this new professional identity, claim the unique authority to judge the meaning of available information cumulatively and translate it into quality clinical practice:

Pharmaceutical representatives constantly visit me, they constantly recommend something, they constantly invite me to some round tables [. . .] They all tell about the same thing, but the decision . . . That’s why we sit there - to make decisions. (GP16)

Overall, physicians in this study maintain that with the intensification of marketing efforts by pharmaceutical companies, characteristic of the ongoing pharmaceuticalization, they have not lost their agency and central role in medical practice and circulation of pharmaceuticals in society. Rather, using connections with the industry via pharmaceutical representatives they have found new ways of staying informed about developments in the rapidly changing pharmaceutical arena, while still playing a crucial role in shaping access of the industry to patients—the ultimate users of pharmaceuticals, through reflexive processing of pharmaceutical information gained from a variety of sources. The engagements between physicians and pharmaceutical representatives are associated with risks, and within the Russian medical community itself there is a diversity of opinions as to where a line should be drawn between legitimate professional relationships and “selling out.” However, according to our informants, a categorical opposition to the physician-industry engagements and attempts to ban it, appear to allot too much power and agency to the pharmaceutical companies and too little to physicians, who now appear to firmly link their professional identity to the ability to weigh and bridge different kinds of knowledge in the context of clinical practice.

## Discussion and conclusion

In this article we examined how physicians engage with complex knowledge and organizational landscapes produced by pharmaceuticalization. By maneuvering the challenges produced by the pharmaceuticalization, physicians find new ways of developing their expertise and of constructing their professional identity, and, thus, of enacting their professionalism. The ways in which they navigate abundant sources of knowledge and use industry resources to overcome constraints of their organizational environment attests to mundane forms of agency exercised by physicians in their relations with industry.

Russian physicians treat representatives as carriers of news about recent developments in the field of drugs and understand their visits as an alerting system, appreciated for its speed and reach. Finding themselves in an environment where circulation of new pharmaceutical knowledge is restricted and enforced clinical standards appear to be informed by considerations other than recent evidence, physicians use a variety of strategies, including receiving and subsequently checking “signals” from the pharmaceutical representatives, to pragmatically navigate this rocky terrain and perform their clinical work. In a context where autonomy of the medical profession is already limited on the structural level by bureaucratic state control, information disseminated by the industry quite paradoxically is used by the physicians to enhance their ground-level agency.

Our informants position themselves as those who, after reflexively bridging different knowledges, are to make decisions in daily clinical practice. Physicians describe the significance of their control over decision-making in such terms that point to the perceived centrality of their role in the pharmaceuticalization process as it is physicians, after all, who select which pharmaceuticals to prescribe. When it comes to the conflicts of interest between the profit-seeking goals of industry and the doctors’ duty to ensure the best possible care for patients, the informants in this study viewed the possibility of such conflicts as connected to the professional competence of each physician. Since they construct professional identity as dependent on the ability to manage different kinds of knowledge, they suggested that it is those who have limited opportunity to exercise this ability are at risk of being too susceptible to pharmaceutical representatives’ messages.

Our study contributes to the scholarship on medical professionalism in a complex organizational environment by providing empirical evidence of what has been conceptually sketched by other authors as a “connective” type of professionalism. Instead of absolutization of professional autonomy this approach suggests viewing professionals as “connected to clients, stakeholders, influences, in such a way that professional identities can be upheld, professional decisions can be taken, and legitimacy can be (re)gained” (Noordegraaf, 2020: 206). In line with this, we offer a nuanced view on professionalism as physicians’ ability to agentically interact with different kinds of knowledge and translate them into clinical work. This study also relates to the pharmaceuticalization scholarship. Previous research has documented the marketing influence of pharmaceutical companies as one of the main drivers of pharmaceuticalization. We illuminate practices physicians employ to resist the stripping away of their agency and to still play a role in shaping pharmaceuticalization processes. This role cannot be reduced to either “giving in” or “resisting” the power of the industry. Physicians attempt to actively and selectively interact with companies, using industry resources to overcome the constraints of the local healthcare settings.

Finally, we do not wish to deny the existence of risks or economic interests associated with the pharmaceutical promotion activities in the arena of medical practice. Previous studies have vividly documented an extensive array of sophisticated and effective practices used by the industry to produce conditions under which specific prescriptions and purchases would be as frequent as possible (Sismondo, 2018). Rather we aspired to zoom in on the practices physicians employ in these circumstances to trace complex engagements between the clinic and industry and to amend what has been called the “neglect of micro-level relations and interactions within many pharmaceuticalization discussions” (Brown et al., 2015: 314). In practice, such an analytical angle can amend the dominant conflict of interest discourse to include attention to related important issues such as the access of physicians to education and professional development opportunities, which are salient in the Russian context.
